# Stratified water columns: homogenization and interface evolution

**DOI:** 10.1038/s41598-024-62035-w

**Published:** 2024-05-20

**Authors:** Mengwei Liu, Junghee Park, J. Carlos Santamarina

**Affiliations:** 1 School of Civil and Environmental Engineering, Georgia Institute of Technology, Atlanta, GA 30332 USA; 2 Department of Civil and Environmental Engineering, Incheon National University, 119 Academy-Ro, Yeonsu-Gu, Incheon, 22012 South Korea

**Keywords:** Ocean sciences, Physical oceanography, Solid Earth sciences, Geophysics

## Abstract

Stratified water columns are often found in lakes and oceans. Stratifications result from differences in density due to salt concentration, temperature, solid content and oxygenation. The stability of stratifications affects bioactivity, sedimentation, contaminant transport and environmental remediation. This study investigates the evolution of 6 stratified water columns created by differences in salinity, suspended minerals and the presence of a bottom heat source. We use acoustic wave reflection, photography, and both electrical conductivity and temperature profiles to track changes in stratification. Results show that multiple concurrent processes emerge across layers in otherwise quiescent water bodies. Dissimilar chemo-thermo conditions give rise to chemical and thermal diffusion, convection, and double-diffusion convection. When stratification involves suspended particles, interlayer processes include diffusiophoresis, flocculation/aggregation, sedimentation, osmosis, and chemo-consolidation; in this case, the specific surface and surface charge of suspended particles, and the salt concentration in contiguous layers determine aggregation-sedimentation-consolidation patterns. The interlayer transition zone acts as a high-pass filter that preferentially reflects low-frequency long-wavelength P-waves; invasive thermal and electrical conductivity probes provide complementary information and may identify stratification even when it is undetected by acoustic signals.

## Introduction

Stratified water bodies often appear in lakes and oceans. Stratifications result from differences in density due to salt concentration (halocline), temperature (thermocline), solid content (minerals and organic—pycnocline), and oxygenation (nutricline). Studies have reported stratification in estuaries (Baltic Sea^1^; Mossel Bay^2^), fjords (Greenland^3^; Petermann^4^), oceanic hot brine pools (Red Sea^5^; Gulf of Mexico^6^; Lake Vanda, Antarctica^7^), underwater hydrothermal flows (Yellowstone Lake^8^; East Pacific Rise^9^), lakes (Dead Sea^10^), deltas (Southeastern Iceland^11^; South China Sea^12^), rivers (Northern California^13^; Krka River, Croatia^14^) and following deep sea tailings disposal (Papua New Guinea^15^).

The stability of stratifications affects bioactivity, sedimentation, contaminant transport and environmental remediation. Non-invasive acoustic waves often enable the rapid detection of stratified water bodies^16–20^. The analysis of wave propagation across interfaces provides the conditions for acoustic mapping in terms of the wavelength, interface transition thickness and the impedance mismatch between layers^21–28^. Field studies use high-frequency acoustic systems to characterize bioactivity^29^, turbulent microstructure^30^ and suspended sediments^31^. However, only a few studies have used acoustic imaging to study interface dynamics in stratified water columns^32,33^.

Furthermore, stratification may emerge due to differences in particle load, salt concentration and temperature; then, the water column may experience phenomena such as double-diffusive convection^34,35^, aggregation and settling, settling-driven convection^36,37^ and layer shearing induced by seafloor currents^38,39^. Extensive previous studies explored particle–particle interaction in water columns^40–46^. However, detailed laboratory studies focused on physio-chemical processes between contiguous layers and across interfaces, and the ensuing evolution of stratification in otherwise quiescent water bodies, are lacking.

This study investigates the evolution of stratified water columns and underlying processes. All tested systems represent quiescent water columns that are gravity-stable due to density differences; thus, they experience no fingering and no shear currents. We use acoustic wave propagation, photography, electrical conductivity and temperature measurements to track the evolution of the stratified systems.

## Experimental study

Experiments include three sets of well-controlled laboratory-scale tests (details in Table 1): (1) thermo-saline driven stratification, (2) saltwater on slurries and (3) light suspensions on saltwater. The experimental design and test protocols are described next.

**Table 1:** Six tested cases: Description, layer properties, and corresponding examples in nature.

	Cases	Water column		Thickness (mm)		Density (kg/m^3^)		P-wave velocity (m/s)		Examples in nature
		Upper	Lower	Upper	Lower	Upper	Lower	Upper	Lower	
Fresh water over brine	1	Fresh water	6 mol/L brine	236	130	1000	1280	1486	1749	Estuaries (Andersson et al.^1^; Roberts et al.^2^) Fjords (Boghosian et al.^3^; Tinto et al.^4^)
	2*	Fresh water	6 mol/L brine	206	160	1000	1280	1486	1749	Red Sea brine pool (Swallow and Crease^5^) Gulf of Mexico brine pool (Wankel et al.^6^) Lake Vanda, Antarctica (Hoare^7^)
Saltwater over dense slurry	3	2 mol/L saltwater	Kaolinite slurry μ = 0.150	306	60	1083	1103	1561	1733	Deep-sea tailings disposal (Shimmield et al.^15^) Underwater plumes (Bouligand et al.^8^; Palmer et al.^9^)
	4	0.25 mol/L saltwater	Bentonite slurry μ = 0.048	211	155	1010	1031	1509	1494	
Light suspension over saltwater	5	Kaolinite suspen μ = 0.002	2 mol/L saltwater	206	160	1002	1083	1604	1561	Light sediment discharge (Stefánsdóttir and Gíslason^11^; Schroeder et al.^12^). Organic colloids in river (Sempéré and Cauwet^14^)
	6	Bentonite suspen μ = 0.002	2 mol/L saltwater	201	165	1002	1083	1548	1561	

*Includes bottom heater to cause DDC. Slurries and suspensions prepared with fresh water.

### Devices

**Figure 1:**
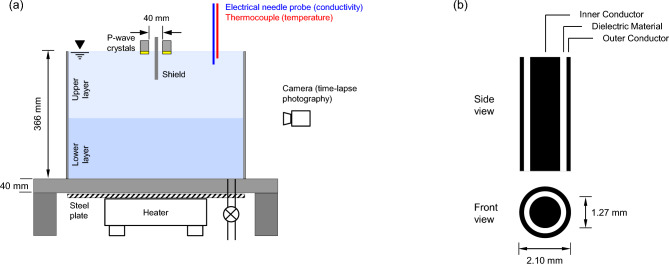
Tank and instrumentation. (**a**) Stratified water column in water tank and peripheral components. (**b**) Needle probe for electrical conductivity profiling.

We use a large-diameter cylindrical water tank to minimize boundary effects (Fig. 1a; plexiglass tank: 500 mm in diameter; 366 mm high; bottom plate: 40 mm thick—See photograph in Supplementary Information 1. The sketch under Supplementary Information 2 shows that lateral wall reflections require significantly longer P-wave travel times than the direct reflections from the liquid–liquid interface). A small hole and an overlain diffuser in the bottom plate allow for controlled fluid injection to create the stratified water column bottom-up (photograph in Supplementary Information 1). The tank sits on a steel plate and heater (used for Case No. 2—Table 1).

We seek to study mm-scale interfaces. Therefore, we select a high damping, water-coupled P-wave transducer pair (wavelength λ ≈ 3 mm; 19 mm diameter V318-SU, Olympus. Calibration and directivity in^47,48^). The frequency response of the source-receiver pair exhibits a main peak at 380-to-420 kHz, with lower peaks between 60-to-120 kHz (Supplementary Information 3). The spacing between the source and receiver crystals is 40 mm (Fig. 1a); a foam shield placed between the transducers prevents acoustic cross-talk.

At predetermined times, we measure the electrical conductivity and temperature profiles for the six stratified systems using a 2.1 mm diameter coaxial termination probe operated at f = 20 kHz to prevent electrode polarization (Fig. 1b—probe details in^49^). The thermocouple attached next to the resistivity probe tip records the temperature profile (relevant to the double-diffusive convection experiment; Case 2—Table 1). The probe is mounted on a motorized linear positioner to enable precise depth control (± 1 mm). The probe advances at 1 mm/s, and remains static at the target depth during the measurement (once the signal displayed on the oscilloscope is stable).

### Sample preparation

We mix NaCl with deionized water to prepare solutions at different concentrations, c = 0.25, 2 and 6 mol/L. Slurries and suspensions are prepared with fresh water and either bentonite (Western sodium bentonite from Euclid’s Pottery Store, USA; liquid limit LL = 360; specific surface S_s_ = 550 m^2^/g) or kaolinite (RP2, Gordon, USA; LL = 78; S_s_ = 21.9 m^2^/g). Suspensions involve a low solids fraction where particles remain in suspension by Brownian motion (water content *w* ~ 100 LL); on the other hand, slurries are stable clay-water mixtures with a water content LL < *w* < 10 LL. The clay slurries make the lower layer in cases No. 3 and No. 4, while the light clay suspensions make the upper layer in cases No. 5 and No. 6 (Table 1).

### Test procedure

We first place the light liquid into the tank, and wait for at least 6 h before injecting the heavy liquid at the bottom; in all cases, there is no “perceptible” movement before the injection of the heavy fluid at the bottom. We monitor the evolution of stratification using P-wave reflections, electrical conductivity and temperature profiles, and time-lapsed photography as applicable. Photographs in the Supplementary Information 4–8 show images for the various cases.

## Results and analyses

This section reports experimental results and analyses for the six stratified water columns described in Table 1. We include additional protocol details that are specific to each case.

### Case 1: fresh water over brine at constant temperature

**Figure 2:**
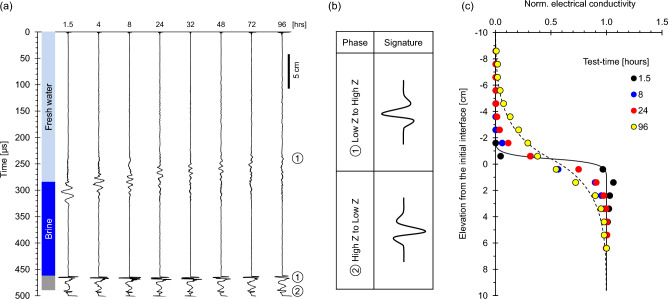
Fresh water over brine at constant temperature: diffusion (Case No. 1—Table 2). (**a**) Reflected P-wave signatures at different test-times (Note: bottom reflector amplitude/10). (**b**) Phase inversion corresponds to the impedance Z = ρ·V mismatch at each interface. (**c**) Conductivity profiles normalized by the brine conductivity.

This first case explores the evolution of liquid interfaces during chemical diffusion when a fresh water layer sits on top of a brine layer (Case No. 1—Table 1; duration = 96 h). Figure 2a presents the reflected P-wave signatures gathered at different times. The freshwater-brine interface provides clear reflections at around 300 μs during the first ~ 48 h. The time to the first arrival and the amplitudes of reflected signals decrease over time. The two reflections at t ≈ 463 μs and 490 μs correspond to the bottom plate (thickness = 40 mm; Fig. 1). The phase of reflected signals is the same for water-brine and brine-plexiglass, but opposite for the plexiglass-air (Fig. 2b), in agreement with the corresponding impedance mismatch. Water-brine reflections have low-frequency contents compared to signals reflected from the plexiglass.

Figure 2c shows normalized electrical conductivity depth profiles. The electrical conductivity exhibits an initially sharp interface which gradually evolves into a diffusion front as salt migrates into the freshwater body above.

### Case 2: fresh water over brine with bottom heat source

The presence of a bottom heat source promotes convection in the lower stratum while there is concurrent heat and ionic diffusion across the liquid–liquid interface; then, a convection cell emerges in the upper layer as well. “Double-diffusive convective” systems have been identified in stratified water columns over heat sources (as early as the 1960’s in the Red Sea^5^).

**Figure 3:**
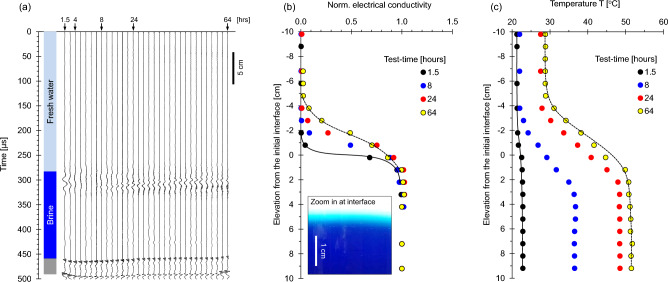
Fresh water over brine with bottom *heatsource*: Double-diffusive convection (Case No. 2—Table 2). (**a**) Reflected P-wave signals at different test-times (Note: bottom reflector amplitude/67). (**b**) Conductivity profiles normalized by the brine conductivity. (**c**) Temperature profiles. The experimental time starts at brine injection (before turning the heater on).

We explore the evolution of a stratified column during double-diffusive convection (Case No. 2—Table 1; duration = 64 h). The P-wave reflections at ~ 300 μs correspond to the freshwater-brine interface; they initially vanish during early diffusion, but resurge once convection starts after turning on the bottom heat source (Fig. 3a; Note: brine injection takes ~ 1.5 h; the heater is turned on right after brine injection—There is no peripheral thermal insulation around the tank).

The lower saltwater layer has some methylene blue to allow for optical observations. The colored layer exhibits a clearly homogeneous intensity in depth and across the interface, in agreement with previous studies that have shown the homogenizing effect of convective circulation within each stratum^32,33^. The normalized conductivity profile readily captures the evolution of the interface (Fig. 3b). Convection homogenizes the ionic concentration and temperature within the convection cell and “shaves” the interface which remains sharp even as diffusion takes place. Consequently, convection preserves short diffusion lengths and increases heat and ionic transport across the interface. Temperature increases homogeneously in the upper layer as well suggesting an upper convective cell (Fig. 3c). The light blue zone in the inset in Fig. 3b captures the transition zone that forms between the freshwater and brine strata.

**Figure 4:**
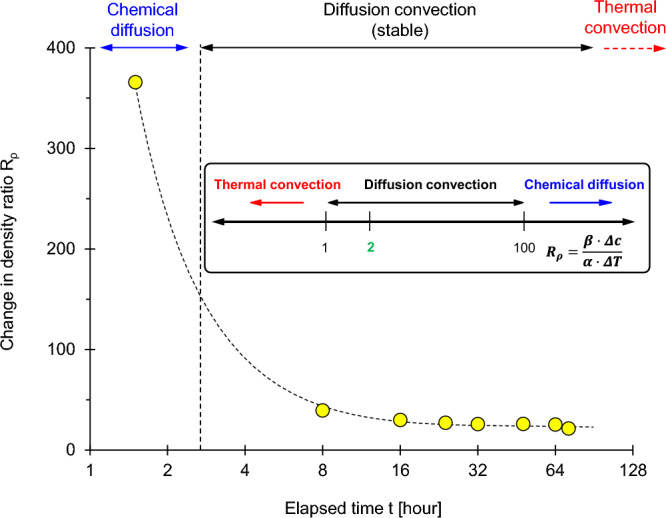
Diffusion, convection and the double-diffusive convection regimes. The plot shows the evolving regimes within the experimental cell. The interpretation is based on the density ratio R_ρ_ as summarized in the inset.

Double-diffusive convection results from the interplay between temperature T [°C] and salt concentration c [mol/L] on the solution density. The ratio between the stabilizer effect of salt concentration Δρ = β·M_salt_·∆c and the convection-promoter effect of temperature Δρ = α·∆T determines the emergence of double-diffusive convection (where ∆c and ∆T are the salt concentration and temperature differences across the transition zone, the thermal expansion coefficient for water is α = 4.1 × 10^–4^ °C^-1^ and the haline contraction coefficient is β = 7.8 × 10^–4^ g/L)^50^: thermal convection prevails if the ratio R_ρ_ = (β·M_salt_·∆c)/(α·∆T) < 1 while double-diffusive convection is dominant if R_ρ_ > 1. The evolution of the R_ρ_ ratio is reported in Fig. 4, starting with pure chemical diffusion, followed by double-diffusive convection and ending with pure thermal convection. In agreement with experimental evidence, conditions for double-diffusive convection start after ~ 8 h when P-wave reflections reappear (Fig. 3a).

### Case 3: saltwater over dense kaolinite slurry

**Figure 5:**
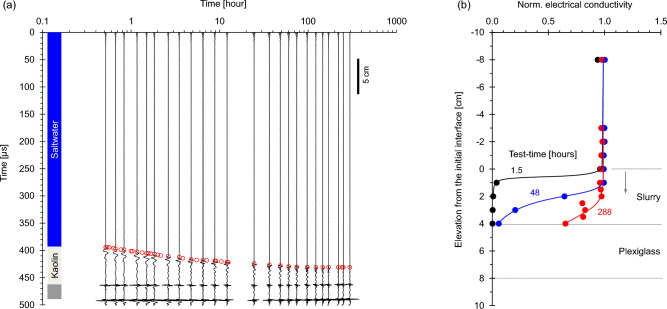
Saltwater over dense kaolin slurry (Case No. 3—Table 2). (**a**) Reflected P-wave signals and kaolinite suspension height obtained from time-lapse images (Note: bottom reflector amplitude: same scale). (**b**) Conductivity profiles normalized by the saltwater conductivity.

Consider a freshwater kaolinite slurry suddenly being overtopped by 2 M saltwater (Case No. 3—Table 1; duration = 300 h). The sequence of P-wave signals in Fig. 5a shows a persistent reflection at ~ 400 μs which corresponds to the saltwater-slurry interface. The coinciding empty red circles show the coinciding interface elevation recovered from time-lapse images. The phase of reflections corresponds to the impedance mismatch at each interface. The kaolinite layer settles as ions diffuse into the slurry causing double layer contraction. The slurry experiences overall densification (the depth-average porosity calculated from the initial water content is 0.938; the change in height implies a porosity reduction to 0.882 after 300 h). Yet, aggregation lowers the stiffness at low effective stress, the impedance mismatch decreases near the interface and reflections decline. The contraction of the kaolinite layer leaves behind low concentration water that mixes with the saltwater above.

The initial electrical conductivity jump across the saltwater-kaolinite interface becomes smoother as salt diffuses into the slurry (Fig. 5b). In this case, the electrical conductivity detects the evolving salt diffusion front into the clay slurry, while P-wave signatures capture the interface migration by layer contraction.

### Case 4: saltwater over dense bentonite slurry

**Figure 6:**
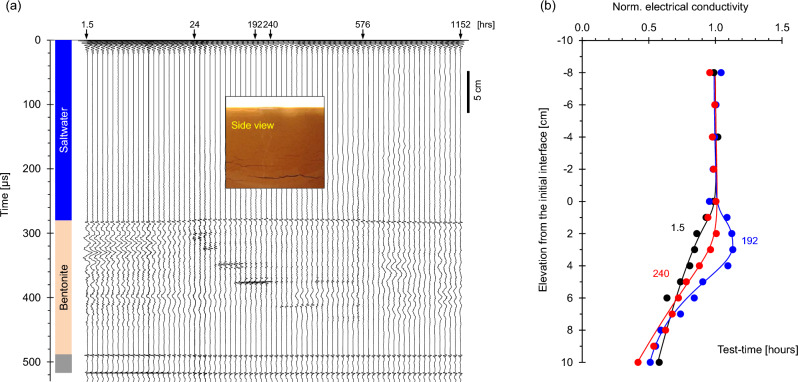
Saltwater over dense bentonite slurry (Case No. 4—Table 2). (**a**) Reflected P-wave signals at different test-times. Reflections from within the slurry layer confirm the emergence of horizontal discontinuities shown on the inset picture (Note: bottom reflector amplitude/50). (**b**) Conductivity profiles normalized by the saltwater conductivity.

This case resembles the previous one, but involves high specific surface bentonite slurry sliding beneath a saltwater layer (Case No. 4—Table 1; duration = 4320 h). Figure 6a shows successive P-wave signals. The saltwater-slurry reflection at ~ 380 μs migrates upwards for the first ~ 600 h before it begins to show layer contraction. This response suggests overall initial swelling by osmotic suction followed by double layer contraction as ions diffuse into the bentonite slurry^51^. Overall, the interface retains its impedance mismatch and produces high frequency reflected signals, i.e., sharp interface.

Reflected P-wave signals and time-lapse images provide evidence of evolving horizontal discontinuities within the bentonite layer and their intermittent downward migration (see insets in Fig. 6a). Vertical discontinuities also form as the diffusion front advances and cause contraction under zero lateral strain conditions.

The electrical conductivity is constant in the upper saltwater layer and decreases with depth below the saltwater-slurry interface (Fig. 6b). Note that the electrical conductivity is quite high in the bentonite slurry prepared with fresh water due to the hydrated counterions and excess salts in the bentonite. Salt diffusion into the bentonite slurry causes a conductivity overshoot at 192 h.

### Case 5: light kaolinite suspension over saltwater

**Figure 7:**
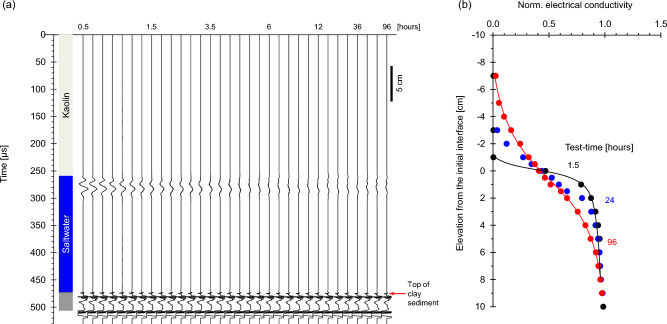
Light kaolin suspension over saltwater system monitoring (Case No. 5—Table 2). (**a**) Reflected P-wave signals at different test-times. There is a reflection from the kaolin sediment above the bottom plate (Note: bottom reflector amplitude/30). (**b**) Conductivity profiles normalized by the saltwater conductivity.

Low solids content freshwater suspensions often reach heavier seawaters in deltaic environments. We explore this situation in the next two cases, starting with low-specific surface kaolinite suspended in fresh water (Fig. 7a; Case No. 5—Table 1; duration = 96 h).

Low frequency reflected P-wave signals from the kaolinite suspension-saltwater interface appear at ~ 260 μs during the first few hours. The amplitude gradually weakens as: (1) ions diffuse upwards and prompt kaolinite flocculation and settlement and (2) diffusiophoresis drives particles in the direction of the concentration gradient and enhances sedimentation^52,53^. The high-frequency reflection at ~ 470 μs shows the sediment layer forming on the bottom plate; the other two reflections correspond to the upper and lower surface of the bottom plexiglass. The phase of reflected signals agrees with the impedance mismatch in each case.

The normalized conductivity shows an initially sharp transition in the stratified column (Fig. 7b), but it smoothens rapidly (compare to Fig. 2b). Apparently, aggregation and sedimentation cause internal currents that promote ion transport.

### Case 6: light bentonite suspension over saltwater

**Figure 8:**
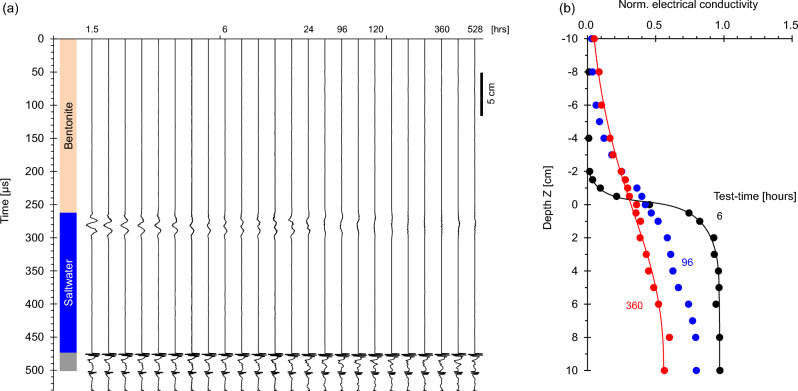
Light bentonite suspension over saltwater system monitoring (Case No. 6—Table 2). (**a**) Reflected P-wave signals at different test-times (Note: bottom reflector amplitude/30). (**b**) Conductivity profiles normalized by the saltwater conductivity.

Finally, let us consider a similar situation to Case 5 but for a suspension made of high specific surface bentonite particles (Case No. 6—Table 1; duration = 528 h). Figure 8a shows low frequency P-wave reflections at ~ 270 μs corresponding to the suspension-saltwater interface. Similar to the kaolinite suspension, upwards ion diffusion and diffusiophoresis cause flocculation and settlement. The settled bentonite is very soft and there is no detectable reflection from the sediment that accumulates at the bottom of the tank.

The initially sharp transition in conductivity across the suspension-saltwater interface rapidly smoothens and conductivity homogenizes in the upper and lower layers (Fig. 8b).

**Figure 9:**
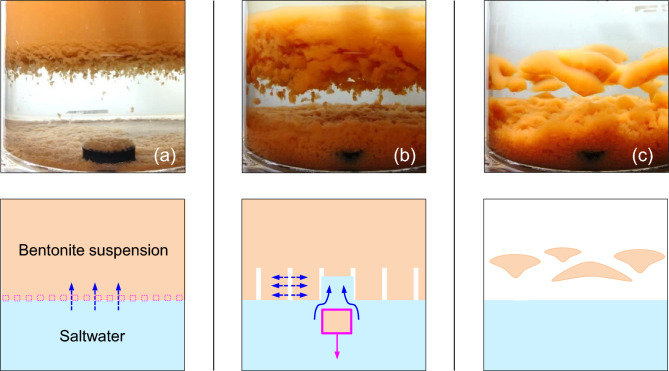
Light bentonite suspension over saltwater—Evolution in time (Case 6—Table 2). Photographs and physical sketches. (**a**) Upward salt diffusion drives flocculation near the interface. (**b**) Flocs fall and saltwater flows up to fill the void. (**c**) Long-permanency remnant bentonite clouds.

Pictures in Fig. 9 show three stages in the evolution of the upper bentonite suspension: (1—Fig. 9a) upward salt diffusion drives flocculation near the interface, (2—Fig. 9b) flocs fall and saltwater flows up to fill the void left by flocs, and (3—Fig. 9c) localized transport and associated currents result in remnant bentonite clouds with long permanency.

## Discussion

### Stratification and interface dynamics—underlying processes

Multiple concurrent processes govern the evolution of stratified water columns and interfaces in quiescent water bodies. The six cases studied above involved chemical and thermal diffusion, convection, diffusiophoresis, flocculation, sedimentation, osmosis and chemo-consolidation (see Table 2).

**Table 2:** Six tested cases: concurrent physical processes inferred from all measurements.

Cases	Stratified layer	Concurrent physical processes
1	Fresh water over brine (without heat)	Salt molecules diffuse into the upper fresh water column gradually smoothing the interface
2	Fresh water over brine (with heat)	Initially, salt molecules diffuse into the upper fresh water column gradually smoothing the interface. Once the heat source starts, convective cells emerge, initially in the lower brine pool; circulation shears and sharpens the interface and homogenizes both temperature and concentration. Convective flow enhances heat and ionic transport across the sharp interface until the whole column homogenizes
3	Saltwater over dense *kaolinite slurry*	Salt molecules diffuse into the lower clay slurry causing densification by double layer contraction and settlement. The lighter low-salinity water left behind mixes with the upper saltwater layer. The saltwater-slurry interface migrates downwards and remains sharp
4	Saltwater over dense *bentonite slurry*	There are two concurrent processes: (1) the bentonite layer rapidly takes water from above and expands towards its equilibrium condition, while (2) salt diffusion eventually causes settlement by double layer contraction. The interface remains sharp at all times
5	*Kaolinite suspension* over saltwater	Salt molecules diffuse into the upper kaolin suspension, kaolinite particles aggregate and fall through the interface eventually forming a sedimentary later above the plexiglass plate. Salt diffusion continues and the interface fades away
6	*Bentonite suspension* over saltwater	Salt molecules diffuse into the upper bentonite suspension, and bentonite particles aggregate into large flocs that fall through the interface. The voids left behind facilitate ion transport; eventually, isolated colloidal clouds remain in suspension. Optically, the interface looks blocky; however, the acoustic impedance smoothens and reflections vanish

Stable stratifications depend on density. The density of aqueous solution *ρ*_*l*_ [gr/cm^3^] is a function of salt concentration c [mol/L], pressure P [MPa] and temperature T [°C]^54^:

1 $${\rho }_{l}={\rho }_{o}+c{\cdot M}_{salt}\left\{0.668+0.44 c\cdot {M}_{salt}+1{0}^{-6}\left[300P-2400 P\cdot c\cdot {M}_{salt}+T\left(80+3T-3300 c\cdot {M}_{salt}-13+47 P\cdot c\cdot {M}_{salt}\right)\right]\right\}$$

On the other hand, the density of a suspension *ρ*_*sl*_ is a function of the liquid density *ρ*_*l*_ and the solids mass fraction *μ* = *m*_*s*_/*m*_*sl*_

2 $${\rho }_{sl}=\frac{{\rho }_{s}}{\mu + {G}_{s}\left(1-\mu \right)}$$

Equations (1) and (2) highlight the role of temperature *T*, salt concentration *c* and solids mass fraction *μ* on layer sequencing and stability. In fact, the “stable” colloidal clouds observed in Case 6 float at an equilibrium position determined by their density *ρ*_*sl*_ and the evolving density of the surrounding liquid *ρ*_*l*_.

The sediment layer that forms immediately above the bottom plate experiences a virtually nil effective stress (Cases 5 and 6). The corresponding asymptotic void ratio *e*_*L*_ varies with soil type and fluid chemistry. The asymptotic void ratio is *e*_*L*_ = 5-to-8 for kaolinite and *e*_*L*_ = 5-to-12 for bentonite sedimented in 2 mol/L saltwater^55,56^. In fact, the specific surface and the asymptotic void ratio are good predictors of suspension stability in stratified water columns.

### Electrical conductivity: solutions and suspensions

The electrical conductivity reflects the volumetric charge concentration and mobility. When the liquid is an ionic solution, the electrical conductivity *σ*_*l*_ [S/m] relies on the motion of hydrated anions and cations^57^; the linear approximation for fully dissociated “strong” electrolytes at low ionic concentration c [mol/m^3^] is:

3 $${\sigma }_{l}=F \sum_{i} {c}_{i} \left|{z}_{i} \right|{ u}_{i}$$

where other parameters are Faraday’s constant *F* = 96,485.3 C/mol, and the ion valence *z* [ ] and mobility *u* [m^2^/(V·s)] for each ionic species (Note: the ionic mobility is the terminal velocity of an ion in an electric field). Colloids in suspension contribute hydrated counterions. Let us assume that hydrated counterions are as mobile as hydrated ions in the bulk fluid (they are not forming inner sphere complexes or within the Stern layer), that particles are sufficiently close to each other or the excitation frequency is sufficiently high so that double layer polarization does not hinder conduction, and that particles do not move at high frequencies. Then, the electrical conductivity of suspensions and slurries *σ*_*sl*_ combines the motion of hydrated ions in the bulk fluid and in the counterion cloud (derivation in Supplementary Information 9—see^58^ for a more comprehensive analysis):

4 $${\sigma }_{sl}=\left(1-{\mu }_{s}\right)\frac{{\rho }_{sl}}{{\rho }_{l}}{\sigma }_{l}+{\mu }_{s}{\rho }_{sl}{S}_{s}{C}_{s}u$$

in terms of the solids mass fraction *μ*_*s*_ = m_s_/m_sl_, mass density *ρ* = m/V [g/m^3^], specific surface *S*_*s*_ [m^2^/g], surface charge density *C*_*s*_ [C/m^2^], and hydrated ion mobility *u* [m^2^/(V·s)]; subscripts relate parameters to phases: *sl* = slurry or suspension, *s* = solid, *l* = liquid. Excess salts in dry clays contribute to the host liquid conductivity.

The mobility of hydrated ions increases with temperature and is hindered by tortuosity in porous media^59,60^. The high conductivity in the lower bentonite slurry and the conductivity overshoot measured at 192 h in Fig. 6b show the combined contributions of ions in solution and the counterion cloud around clay surfaces.

### Wave propagation velocity—bottom ‘lift-up’ effect

The arrival time for reflections returning from the bottom plexiglass plate decreases during diffusion (Cases 1 & 2); this “lift-up” effect indicates a higher overall velocity as diffusion-driven homogenization takes place. Let us consider a layer of thickness *x*_*1*_ and salt concentration *c*_*1*_ on top of a layer of thickness *x*_*2*_ and salt concentration *c*_*2*_. The P-wave velocity is a linear function of salt concentration *V* = *V*_*1*_ + *n⋅Δc* for small changes in salt concentration *Δc*^61^. Then, the travel time *t*_*str*_ across the two strata is:

5 $${t}_{str}=\frac{{x}_{1}}{{V}_{1}}+\frac{{x}_{2}}{{V}_{1}+n\left({c}_{2}-{c}_{1}\right)}$$

After diffusive homogenization, the travel time *t*_*hom*_ becomes:

6 $${t}_{hom}=\frac{{x}_{1}+{x}_{2}}{{V}_{1}+n\left(\frac{{c}_{1}{x}_{1}+{c}_{2}{x}_{2}}{{x}_{1}+{x}_{2}}-{c}_{1}\right)}$$

It can be shown that t_hom_ ≤ t_str_ for all field conditions; therefore, “lift-up” should be expected in haline stratification, even in the absence of interface reflections.

### Wave propagation across interfaces in stratified water columns

**Figure 10:**
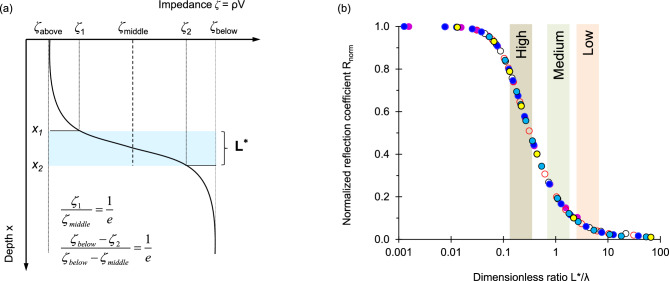
Wave propagation across a smoothly varying interface. (**a**) Assumed impedance profile *ζ* versus depth x. Adopted characteristic length L* of the transition zone (shaded blue area). (**b**) Numerically computed normalized reflection coefficient R_norm_ as a function of the transition length L* normalized by the signal wave length λ—Multiple realizations for various field conditions. The three bands superimposed on the plot represent experimental results (amplitudes referenced to bottom reflections).

Reflections from the tested liquid interfaces exhibit time varying frequency content and/or amplitude. We conduct 1-D numerical simulations to gain an understanding of wave propagation across layers with a gradual transition zone. Figure 10a shows a schematic of the impedance profile versus depth *ζ*(Z). We normalize the reflection coefficient (determined as a ratio between the reflected and incident energy) dividing it by the maximum reflection coefficient defined by the impedance mismatch for L*/λ = 0 where L* is the characteristic transition length and *λ* is the wavelength of the input signal. There are two distinct asymptotic trends in Fig. 10b: *R*_*norm*_ → 1 as L*/*λ* → 0, and* R*_*norm*_ → 0 when L*/λ → ∞. These results indicate that reflections decrease for more gradual changes in impedance in thicker interface transitions L* (for a given frequency and wavelength) and that the transition zone acts as a high-pass filter that preferentially reflects low-frequency long-wavelength signals.

These results explain the relatively low frequency component *f* ≈ 60 kHz in reflections returned from thick L* interlayer transition zones (Case 1 and 2 in Figs. 2a and 3a; Case 3 in Fig. 5; Case 5 in Fig. 7; Case 6 in Fig. 8—see the power spectrum of the piezo crystal pair in Supplementary Information 3). Furthermore, note that the amplitude of the reflected signal from the fresh water-brine interface decreases with time as the diffusion length increases L* (Fig. 2a).

The three bands superimposed on Fig. 10b represent reflection amplitudes referenced to bottom reflections for measurements gathered in this study (Cases 1, 2, and 5). Clearly, both impedance mismatch among contiguous layers and the thickness of the interlayer transition zone—captured in the dimensionless ratio L*/λ—determine our ability to detect stratified water layers using marine seismic.

## Conclusions

Stratified water columns appear in lacustrine, deltaic and marine environments. The underlying differences in density result from ionic concentration (halocline), temperature (thermocline), mineral suspensions (pycnocline) and bioactivity (nutricline). Results from this study show that multiple concurrent, coupled processes emerge across layers in otherwise quiescent water bodies, and determine the evolution of stratification.

- Dissimilar chemo-thermo conditions give rise to chemical and thermal diffusion and convection. The presence of a bottom heat source may trigger double-diffusive convection. Convection cells sustain a sharp thermal and chemical interface, but quicken the overall homogenization of the water column.
- Seasonal sediment loads may produce light freshwater suspensions that sit on top of saltwater layers, or high-solids content slurries that sink beneath the seawater column. In this case, the specific surface and surface charge of suspended particles, and the salt concentration in contiguous layers determine aggregation-sedimentation-consolidation patterns.
- When low-solid suspensions sit on saltwater, upward ion diffusion into the suspension promotes diffusiophoresis and increases the sedimentation rate. When high specific surface clay particles are involved, physio-chemical interaction-induced aggregation, floc sedimentation and contraction-driven discontinuities further enhance settling rates; yet, isolated high specific surface colloidal clouds may remain buoyant within the water column with long permanency.
- On the other hand, when high solid content slurries sink beneath the saltwater, downward ion diffusion into the slurry produces chemo-mechanically coupled osmotic and double-layer contraction effects. Chemo-consolidation releases freshwater and may cause horizontal opening-mode discontinuities in high specific surface area clayey sediments (e.g., bentonite); these horizontal discontinuities migrate downwards together with the diffusion front. There is chemo-consolidation in low specific surface area clays as well (e.g., kaolinite); however higher permeability allows for excess pressure dissipation and ionic diffusion does not cause open mode discontinuities.
- The remote detection and monitoring of stratified water columns with acoustic waves are limited by impedance mismatch, and the ratio between the interlayer transition thickness and the wavelength, L*/*λ*: the interface becomes invisible when the transition zone is thick and L*/*λ* > >  0.1, i.e., the interface acts as a high-pass filter. Invasive thermal and electrical conductivity probes provide complementary information and may identify stratification even when it is undetected by acoustic signals.

### Supplementary Information

Supplementary Information.

## Data Availability

Data sets for this research are available at Figshare: 10.6084/m9.figshare.23928945.v1.

## Abbreviations

*c* [mol/L]: Ionic concentration
*C*_*s*_ [C/m^2^]: Surface charge density
*e*: Void ratio (*e*_*L*_: asymptotic void ratio at low effective stress)
*f* [Hz]: Frequency
*F* [C/mol]: Faraday’s constant
*G*_*s*_: Specific gravity
*L** [mm]: Characteristic diffusion length
*LL* [%]: Liquid limit
*m* [gr]: Mass (subscript: sl = slurry or suspension; s = solid; l = liquid)
*M*_*salt*_ [gr/mol]: Salt molar mass
*n*: Salt concentration-dependent P-wave velocity factor
*P* [MPa]: Pressure
*R*_*norm*_: Normalized reflection coefficient
*S*_*s*_ [m^2^/gr]: Specific surface area
*t* [s]: Time (subscript: str = stratified; hom = homogeneous)
*T* [^°^C]: Temperature
*u* [m^2^/(V·s)]: Ion mobility (subscript: ci = counterions)
*V* [m/s]: P-wave velocity (subscript: o = freshwater)
*x* [m]: Length
*w* [%]: Water content
*z*: Ion valence
ζ [kg/(m^2^·s)]: Impedance
λ [mm]: Wave length
*α* [1/^°^C]: Thermal expansion coefficient; for water: α = 4.1 × 10^–^^4^ °C^−^^1^
*β* [L/g]: Haline contraction coefficient β = 7.8 × 10^–^^4^ L/g
*ρ* [kg/m^3^]: Mass density (subscript: sl = slurry or suspension; l = liquid; o = freshwater)
*µ*_*s*_: Solids mass fraction = mass solids/mass suspension
*σ* [S/m]: Electrical conductivity (subscript: sl = slurry or suspension; l = liquid)
*Δ**c* [mol/L]: Change in salt concentration
*ΔT* [°C]: Change in temperature
*Δ**ρ* [kg/m^3^]: Change in mass density
