# Supplementary material for: Stratified water columns: homogenization and interface evolution
Source: Sci Rep. 2024 May 20;14:11453. doi: 10.1038/s41598-024-62035-w (PMC11106328; doi:10.1038/s41598-024-62035-w)
Supplement: Supplementary file 1 — Supplementary Information. [file 41598_2024_62035_MOESM1_ESM.pdf]

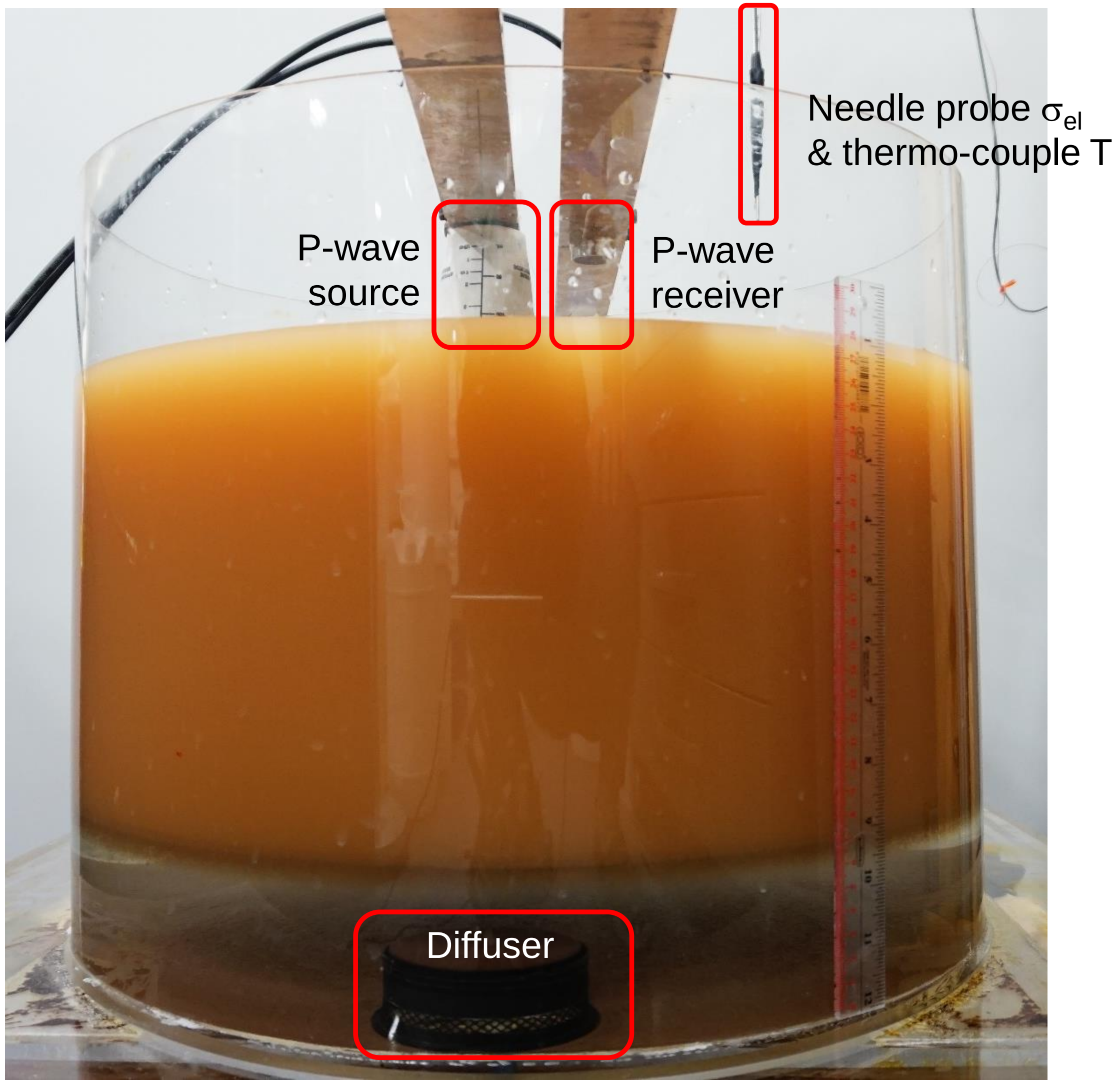

Supplementary Information 1. Experimental set-up. The peripheral foam insulation around the P-source prevents cross talk. The diffuser above the injection point allows horizontal fluid invasion at low flow rate.

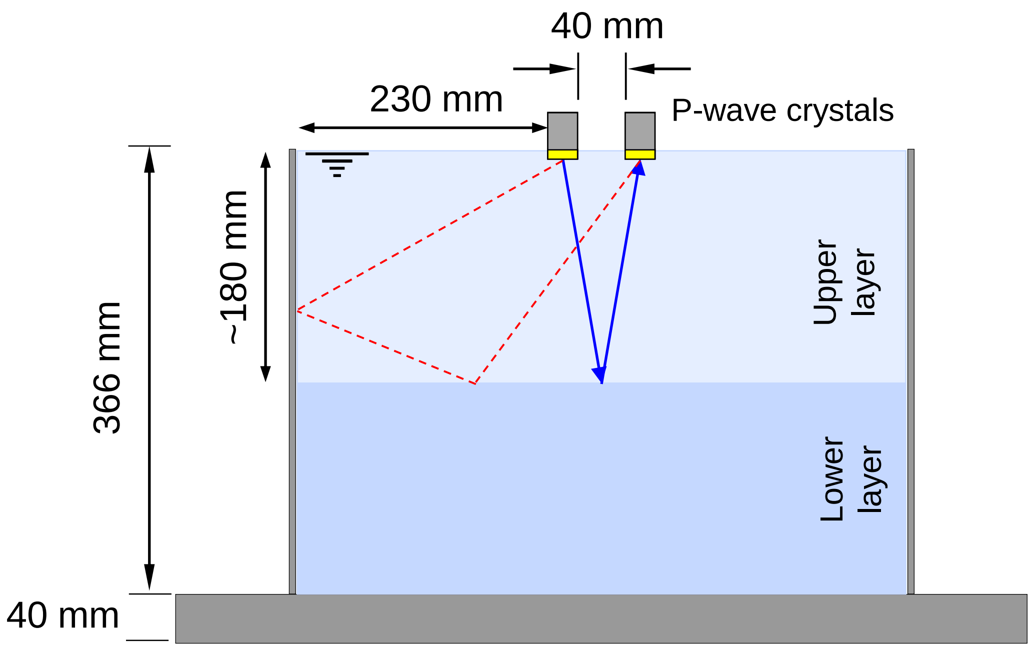

Supplementary Information 2. Wall effect: A Snell-Pythagoras based analysis readily shows that lateral wall reflections require much longer travel times than direct reflections from the liquid-liquid interface (~280 µs in the sketched case)

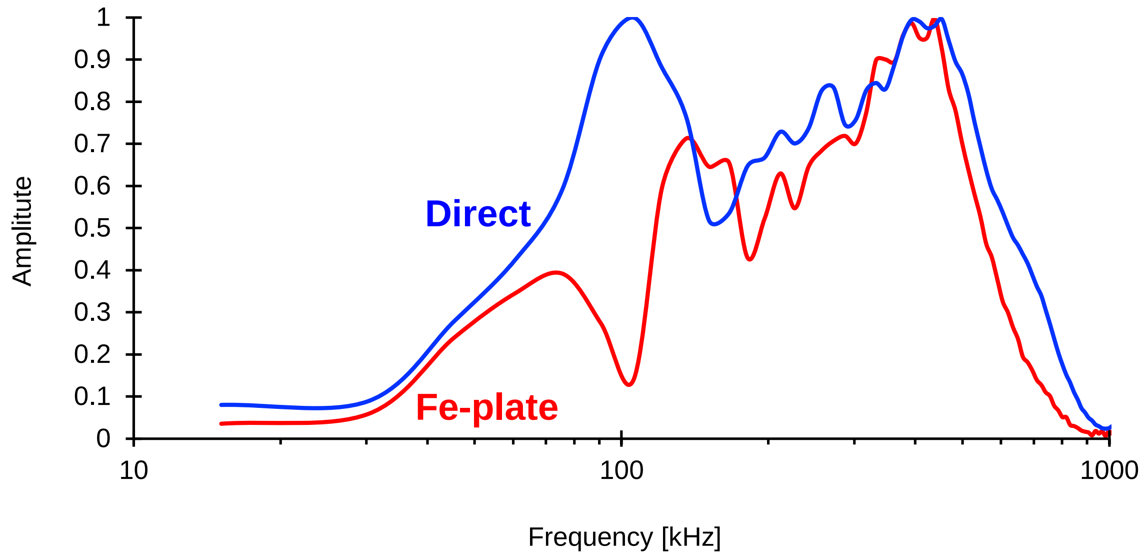

Supplementary Information 3. Piezo-crystal pair: Frequency response. Input signal: Step function. Medium: single layer water column. Blue: Spectrum based on direct wave (submerged and aligned crystals). Red: Spectrum based on wave reflected from a thick steel plate at the bottom.

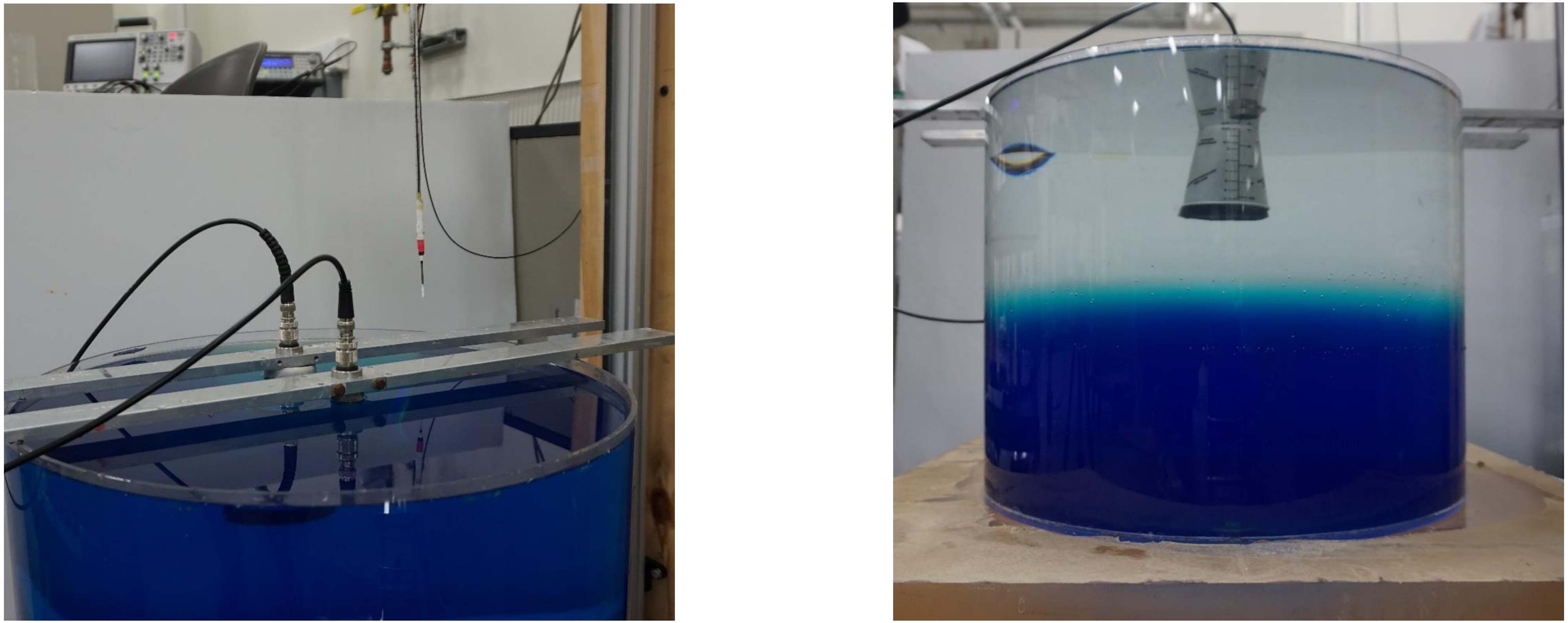

Supplementary Information 4. Fresh water over brine. Left: at constant temperature (Case No. 1). Right: with heat source to create DDC conditions (Case No. 2).

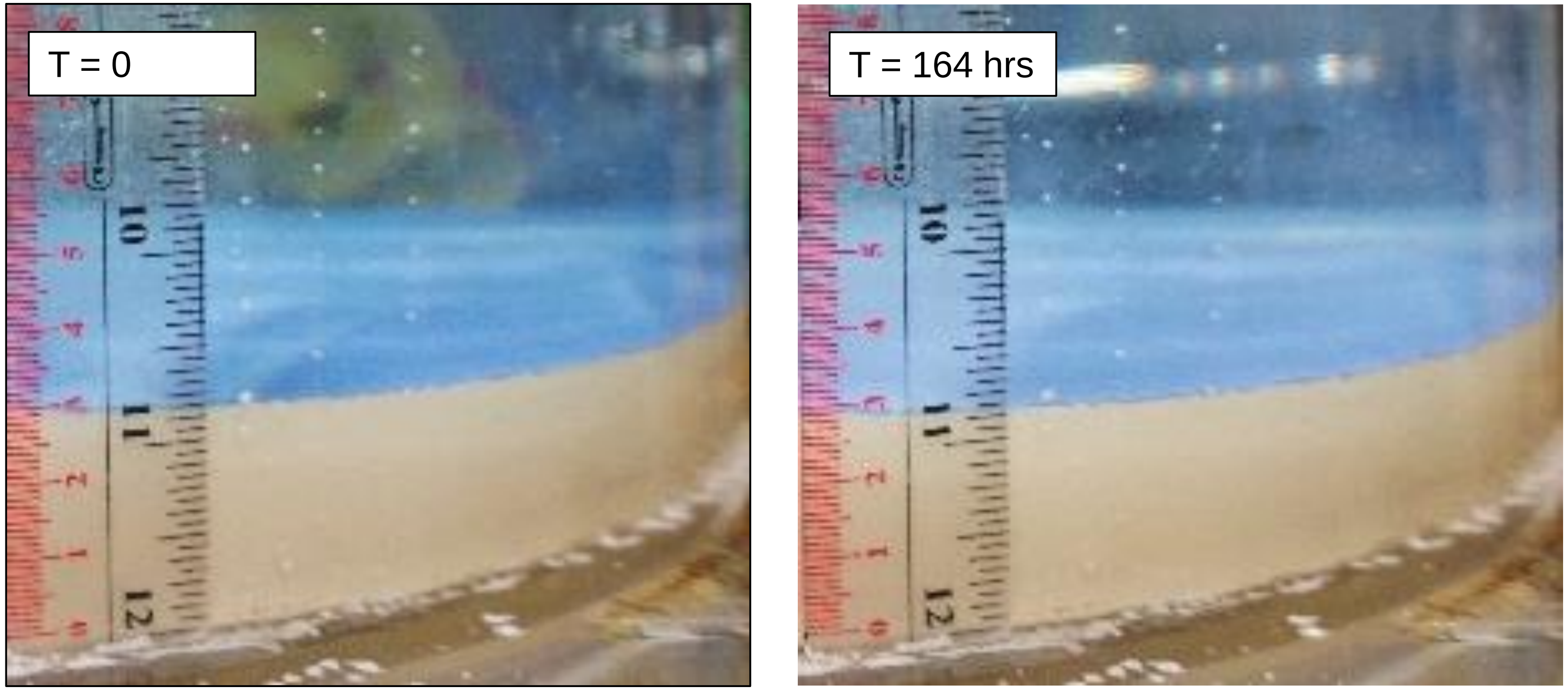

Supplementary Information 5. Saltwater over dense kaolinite slurry (Case No. 3)

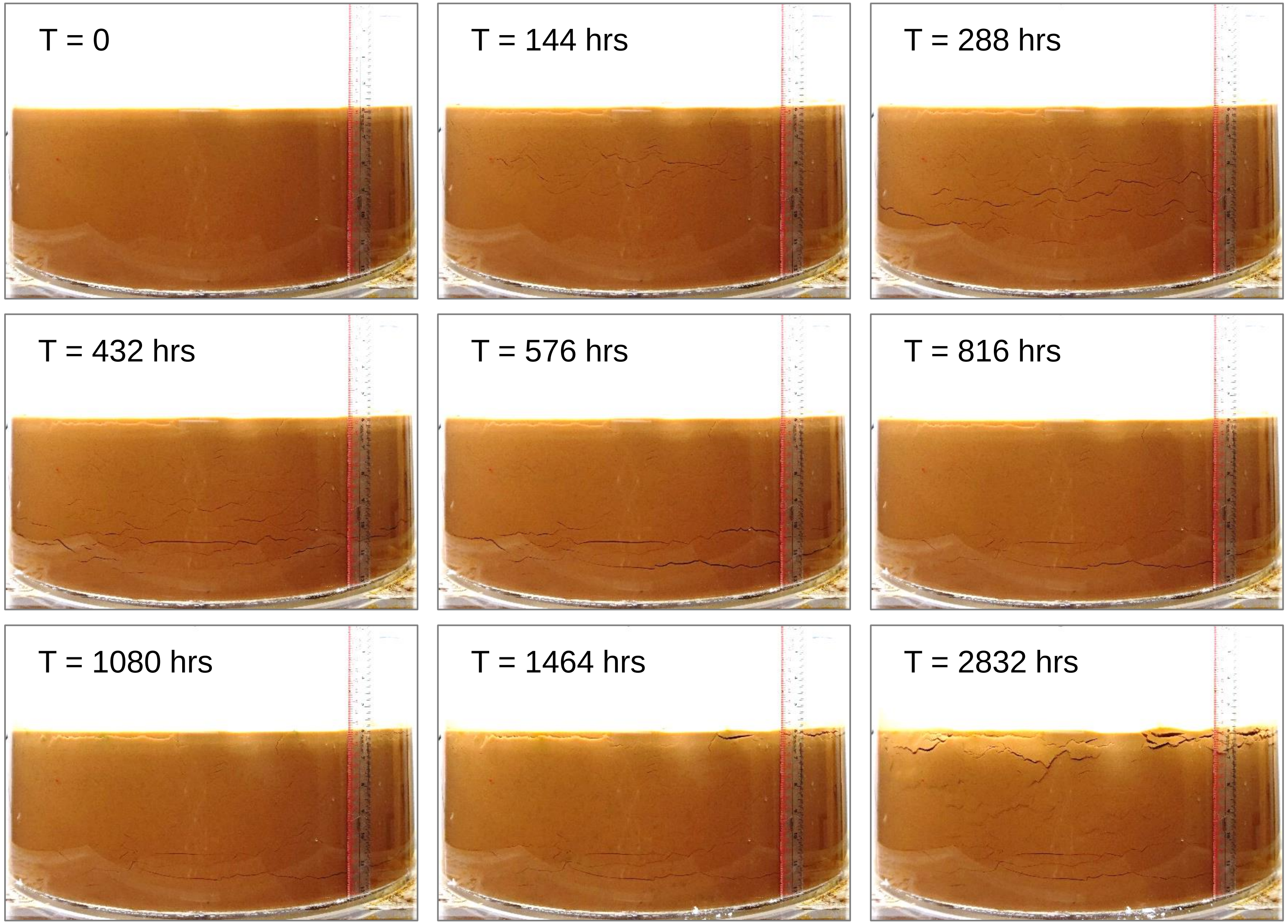

Supplementary Information 6. Saltwater over dense bentonite slurry (Case 4)

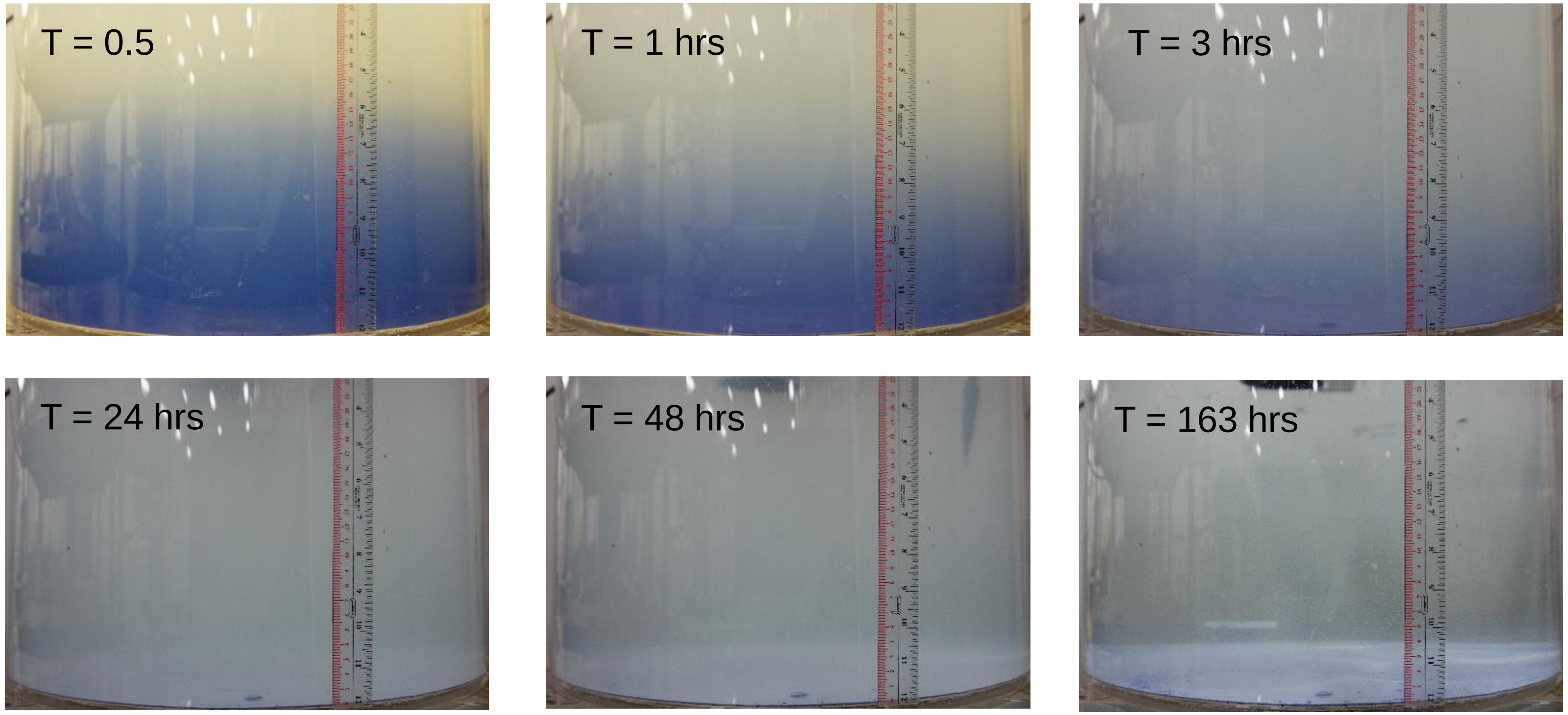

Supplementary Information 7. Light kaolinite suspension over heavier brine (Case 5)

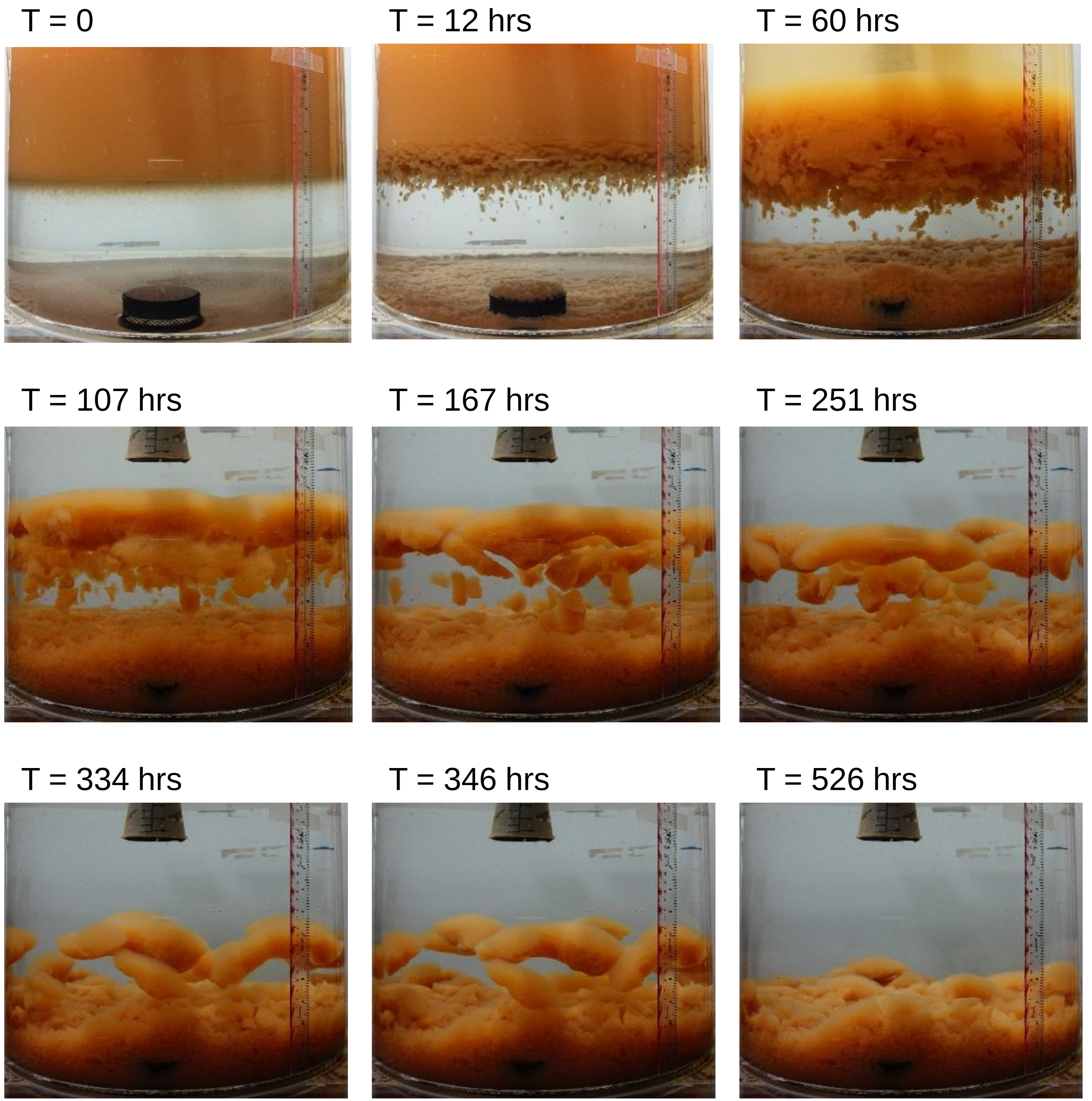

Supplementary Information 8. Light bentonite suspension over heavier brine (Case 6)

Conductivity of suspensions and slurries

$$\sigma_{mix} = \frac{\sigma_l \cdot V_l + M_s \cdot S_s \cdot C_s \cdot u}{V_{sl}}$$ effective surface charge C_s (mobile)

In terms of porosity $n = \frac{V_l}{V_{sl}}$

$$\sigma_{mix} = \frac{\sigma_l \cdot V_l + V_s \cdot \rho_s \cdot S_s \cdot C_s \cdot u}{V_{sl}} \qquad \rho_s = \frac{M_s}{V_s}$$

$$\sigma_{mix} = \sigma_l \cdot n + (1-n) \cdot \rho_s \cdot S_s \cdot C_s \cdot u$$

In terms of mass fraction of soils $\mu_s = \frac{M_s}{M_{sl}}$

$$n = \frac{V_l}{V_{sl}} = \frac{M_l}{M_{sl}} \cdot \frac{\rho_{sl}}{\rho_l} = \frac{M_{sl} - M_s}{M_{sl}} \cdot \frac{\rho_{sl}}{\rho_l} = (1-\mu_s) \cdot \frac{\rho_{sl}}{\rho_l}$$

$$\frac{M_s}{V_{sl}} = \frac{M_s}{M_{sl}} \cdot \rho_{sl} = \mu_s \cdot \rho_{sl}$$

$$\sigma_{mix} = (1-\mu_s) \cdot \frac{\rho_{sl}}{\rho_l} \cdot \sigma_l + \mu_s \cdot \rho_{sl} \cdot S_s \cdot C_s \cdot u$$

Note: to relate mass fraction to water content

$$\omega = \frac{M_l}{M_s} = \frac{M_{sl} - M_s}{M_s} = \frac{1}{\mu_s} - 1$$

Suspension: ω = 100 LL = 360

Slurry: ω = 5 LL = 18

Examples

Faraday constant Fa = 96485 C/mol

Salt water c = 0.25 mol/L v = 1 u = 6×10-8 $m^2/s \cdot V$

Na = 5.2 Cl = 7.9

σ_l = 2 × (Fa · c · v · u) = 2.895 S/m (Note: multiplied (×2) to include Na and Cl)

Deionized water with bentonite

σ_l = 0 S/m ρ_l = 103 kg/m^3

S_s = 550 m^2/gr C_s = 0.1 C/m^2

Suspension: μ_s = (360 + 1)-1 μ_s = 0.003 ρ_{sus} = 1003 kg/m^3

$\sigma_{sus} = \mu_s \cdot \rho_{sl} \cdot S_s \cdot C_s \cdot u$ σ_{sus} = 0.009 S/m

Slurry: μ_s = (18 + 1)-1 μ_s = 0.053 ρ_{slu} = 1021 kg/m^3

$\sigma_{slu} = \mu_s \cdot \rho_{sl} \cdot S_s \cdot C_s \cdot u$ σ_{slu} = 0.177 S/m

Supplementary Information 9. Electrical Conductivity – Suspensions and Slurries
